# RResolver: efficient short-read repeat resolution within ABySS

**DOI:** 10.1186/s12859-022-04790-z

**Published:** 2022-06-21

**Authors:** Vladimir Nikolić, Amirhossein Afshinfard, Justin Chu, Johnathan Wong, Lauren Coombe, Ka Ming Nip, René L. Warren, Inanç Birol

**Affiliations:** 1 Canada’s Michael Smith Genome Sciences Centre at BC Cancer, 570 W 7th Ave, Vancouver, V5Z 4S6 Canada; 2 The University of British Columbia, 2329 West Mall, Vancouver, V6T 1Z4 Canada

**Keywords:** Short reads, *De novo* assembly, Repeat resolution, Scalable, Bloom filters

## Abstract

**Background:**

*De novo* genome assembly is essential to modern genomics studies. As it is not biased by a reference, it is also a useful method for studying genomes with high variation, such as cancer genomes. *De novo* short-read assemblers commonly use de Bruijn graphs, where nodes are sequences of equal length *k*, also known as k-mers. Edges in this graph are established between nodes that overlap by $k - 1$ bases, and nodes along unambiguous walks in the graph are subsequently merged. The selection of *k* is influenced by multiple factors, and optimizing this value results in a trade-off between graph connectivity and sequence contiguity. Ideally, multiple *k* sizes should be used, so lower values can provide good connectivity in lesser covered regions and higher values can increase contiguity in well-covered regions. However, current approaches that use multiple *k* values do not address the scalability issues inherent to the assembly of large genomes.

**Results:**

Here we present RResolver, a scalable algorithm that takes a short-read de Bruijn graph assembly with a starting *k* as input and uses a *k* value closer to that of the read length to resolve repeats. RResolver builds a Bloom filter of sequencing reads which is used to evaluate the assembly graph path support at branching points and removes paths with insufficient support. RResolver runs efficiently, taking only 26 min on average for an ABySS human assembly with 48 threads and 60 GiB memory. Across all experiments, compared to a baseline assembly, RResolver improves scaffold contiguity (NGA50) by up to 15% and reduces misassemblies by up to 12%.

**Conclusions:**

RResolver adds a missing component to scalable de Bruijn graph genome assembly. By improving the initial and fundamental graph traversal outcome, all downstream ABySS algorithms greatly benefit by working with a more accurate and less complex representation of the genome. The RResolver code is integrated into ABySS and is available at https://github.com/bcgsc/abyss/tree/master/RResolver.

**Supplementary Information:**

The online version contains supplementary material available at 10.1186/s12859-022-04790-z.

## Background

*De novo* genome assembly has a wide range of applications, such as gene annotation [1], phylogenetic inference [2], identifying polymorphisms [3] and structural variations [4]. *De novo* assembly specifically is used when either no reference genome is available, or to avoid the biases that may be introduced by using one. For example, a reference genome will not be available when sequencing and annotating the genome of a species for the first time. Another example where *de novo* genome assembly is of prime importance is in cancer studies, in which structural differences between the sequenced tumor and the reference are important.

Many short-read *de novo* assemblers use a de Bruijn Graph (DBG) based approach [5–10]. DBGs are directed graphs defined on an alphabet *S* and node size *k*, where all of the nodes are composed of *k* sized strings containing the characters from the alphabet. For every pair of nodes *x* and *y* there is a directed edge going from *x* to *y* if the $k - 1$ suffix of *x* is equal to the $k - 1$ prefix of *y*, i.e., they overlap by $k - 1$ symbols. In graph theory, a DBG has a node for every permutation of *S* symbols. In the genome assembly problem, however, a variant is used wherein the nodes are all read substrings of size *k*, known as k-mers, and the valid symbols are $S = \{A, C, T, G\}$. The assembly process usually starts by splitting all reads into k-mers and storing them in a data structure, typically a hash table [11]. This allows node adjacency to be queried in constant time, as opposed to searching for overlaps. In recent years, the use of more succinct and resource-efficient data structures in *de novo* genome assemblers, such as Bloom filters, have increased in popularity [5, 6].

A Bloom filter [12] is a probabilistic data structure that has the operations of a set: insertion of an element and querying for the presence of an element. The set is typically implemented as a bit vector initialized with all zeroes. On insertion, the element is hashed into a predetermined number of hash values, *h*, which represent indices in the bit vector where the bits get set. Essentially, the content of the element is compressed into only *h* bits, making Bloom filters very memory efficient. To query for the presence of an element, the element is also hashed into *h* values and those are used as indices into the bit vector to check if the bits are set. Because the bit vector is limited in size, some of the bit indices from different elements may overlap. This can produce a false positive if the queried element bit indices happen to land on indices of other previously inserted elements, even if the queried element has never been inserted. The chance of bit index overlap between elements and thus false positives is increased with the reduction in size of the bit vector. In this way, Bloom filters allow the user to make the trade-off between memory usage and false positives. Note that false negatives are not possible in a Bloom filter designed this way, as once an element is inserted, its set bit indices stay unchanged. Since Bloom filters are highly memory efficient, they have been widely used with memory intensive genomic data [5, 6, 13, 14] and are also used in this work for memory usage scalability.

Repetitive sequences are one of the main confounders of genome assembly. If the same DNA sequence is repeated at a single locus, potentially many times, it is known as a Tandem Repeat (TR). Otherwise, if the same sequence appears at different loci across the genome, as Transposable Elements (TEs) do, it is an interspersed repeat. In the context of DBG based assembly, a repeat that is at least $k - 1$ bases long will create a false edge as any sequence overlap of that length creates an edge. While constructing a DBG, it is impossible to disambiguate repeats that are $k - 1$ bases or longer, and this task is left to the downstream stages of the assembly process.

To illustrate the magnitude of the problem repeats pose to genome assembly, it has been estimated that half or more of the human genome is comprised of repeats [15]. The typical length of a TE is on the order of several kbp, ranging up to 20 kbp in eukaryotes [16]. A third of mammalian genomes consist of TEs and in vertebrates such as zebrafish they make up more than half of the genome [17]. Since current short-read lengths are in the 100–300 bp range [18], they are unable to span a large number of TEs. On the other hand, TRs can have motifs as short as 1 bp. While the motif may be fully spanned by a short read, the number of repetitions may not be possible to estimate with short reads alone. The multiplicity of the motif in the sequencing data is also not a reliable clue to the number of repetitions as the reads are not evenly distributed across the sequenced genome.

Due to non-uniform genome read coverage in the sequencing data [19], regions of the genome with less short-read coverage will have more sparse overlaps between reads, whereas a highly covered region will have an abundant number of reads that have significant overlap. This is where the choice of *k* comes into play—a smaller size will capture the overlap in both low and high coverage regions, but will additionally include many spurious overlaps due to repeats, complicating the graph. On the other hand, a larger size will reduce the number of spurious overlaps but genuine overlaps from less covered regions will also be missed. To overcome this issue, some *de novo* assemblers, such as SPAdes [8], IDBA [20], SOAPdenovo2 [9], and MEGAHIT [21], use an array of *k* values, starting from a small *k* to achieve high connectivity and then proceed to untangle the graph with higher *k* values. These methods demonstrate improved assembly quality, but they have been limited to small *k* value increments or multiple DBG constructions. This is problematic for large genomes (e.g., human), where the assembly graph is large and iterating over a number of *k* values may significantly inflate the run time. There is also room for improvement in the span of *k* that is utilized, as it is not efficient to reach a high *k* value with small steps.

To address these issues, we developed RResolver, a tool for resolving junctions in the assembly graph. The tool utilizes additional short-read information in a scalable manner by taking a larger *k* value than the one used to construct the initial DBG in order to resolve junctions caused by sequence repeats. This larger *k* step bypasses multiple short *k* increments, thus reducing the overall run time, but comes with a set of challenges that have been explored in this study. The initially constructed DBG is worked on directly, without the need for any costly graph reconstruction steps. Additionally, to minimize memory usage, a Bloom filter is employed for k-mer storage. Here we show how RResolver helps improve both the contiguity and accuracy of ABySS assemblies, and demonstrate that it scales well to large genomes. Additionally, we show how ABySS with RResolver compares to other leading genome assemblers on human and *E. coli* sequencing data.

## Results

### Algorithm overview

Herein, DBG assembly *k* is denoted as $k_{assembly}$ and the larger *k* used by RResolver as $k_{rresolver}$. To improve a given DBG assembly, RResolver attempts to find k-mers of size $k_{rresolver}$ along assembly graph paths surrounding a repeat in order to evaluate their correctness. First, all k-mers of size $k_{rresolver}$ bases are extracted from the reads and stored in a Bloom filter [12] for efficient memory use. To find $k_{rresolver}$ k-mer counts along a path, a sliding window of size $k_{rresolver}$ is used, querying the Bloom filter for presence or absence at every step with a step size of 1bp.

Additional file 1: Fig. S1 shows an example in which junction paths can be examined. All paths of three nodes in length are evaluated; in Additional file 1: Fig. S1, that would mean all possible paths from nodes to the left to the nodes to the right: ARX, ARY, ..., CRZ. The algorithm is applied to every repeat short enough for the sliding window to span the whole repeat node sequence, overlap the nodes adjacent to the repeat along the path in question, and to perform a sufficient number of tests (sliding window moves). The number of tests is dynamically determined based on the number of expected $k_{rresolver}$ k-mers (based on local sequencing coverage, assuming the path is correct) along each tested path.

$k_{rresolver}$ sized k-mers found are tallied for every path and the paths where the k-mer count is below a threshold are considered unsupported and hence removed from the graph. Any unambiguous paths resulting from this resolution have their nodes merged, with each path getting its own copy of the repeat sequence.

#### False positives

As a probabilistic data structure, Bloom filters may return false positives on query operations. To deal with these false positives when considering path support, a threshold is set for the number of $k_{rresolver}$ k-mers that need to be found along a path for it to be considered supported. A sufficiently high threshold tolerates a number of false positive matches in the Bloom filter before considering a path supported.

The Bloom filter False Positive Rate (FPR) increases with the number of stored elements, therefore the number of $k_{rresolver}$ k-mers inserted should be minimized. On the other hand, storing more $k_{rresolver}$ k-mers increases the chance that correct paths are identified. To compromise between these observations in RResolver, the number of stored k-mers per read is equal to the support threshold. This effectively allows one read found along a path sufficient to consider that path supported.

The number of false positives depends on a few factors, such as the number of tests done per path (which depends on read coverage), the number of possible paths, and the FPR of the Bloom filters. The FPR of the Bloom filter is modulated with the available memory budget and as RResolver is used alongside a short-read assembler, we can assume that it has the same memory constraints. As shown later in Performance assessment subsection, RResolver can work with tight memory constraints alongside the low memory footprint assembler ABySS.

#### Varying coverage

Read coverage may fluctuate across the genome [19], thus the number of $k_{rresolver}$ k-mers expected along each path may vary. In order to reliably determine whether a path is supported, RResolver calculates the number of tests required to find a sufficient number of $k_{rresolver}$ k-mers along a path to pass the support threshold.

Given $k_{assembly}$ k-mer coverage of a graph node, i.e., the sum of multiplicities of all the $k_{assembly}$ k-mers that comprise the node, provided by the assembler, the expected number of $k_{rresolver}$ k-mers is found proportionally. Since every read provides $l - k_{assembly} + 1$ many k-mers of length $k_{assembly}$, where *l* is read length, the number of reads that have contributed to the node in question can be determined. Given the number of reads in a node and the length of that node, the approximate number of bases between subsequent reads is calculated as the node length over the number of reads. To find a read along a path, on average, the sliding window should move the number of bases equal to the average number of bases between reads. As each read can provide a number of $k_{rresolver}$ k-mers, the sliding window moves an extra number of bases equal to the number of $k_{rresolver}$ k-mers extracted per read in order to capture all of them.

Estimating coverage allows the algorithm to skip less covered regions of the graph where $k_{assembly}$ has been an appropriate choice and further increase in *k* size is not helpful. The criteria to skip a region is simply, if the number of required tests is greater than the possible number of moves the window can do, given the repeat and sliding window sizes. For a sliding window, there are only so many moves it can perform while still overlapping all three nodes that form the path in question, giving an upper limit on the number of tests that can be done in a repeat. If any path in a tested repeat is found to have low coverage such that doing a sufficient number of sliding window moves is impossible, the whole repeat is skipped. Despite possibly knowing whether other paths are supported, the repeat as a whole cannot be resolved accurately without complete information and trying to resolve it could lead to misassemblies.

#### Complex repeats

In highly repetitive regions, the graph becomes particularly complex. The incoming and outgoing nodes from the tested repeat can be repeats themselves, and are often quite short. This can result in the sliding window being longer than the three nodes that are considered as a path. In such cases, the nodes that branch out of the incoming and outgoing nodes are also taken to be possible segments of the path, as shown in Additional file 1: Fig. S2. Branching is done to the extent to which is needed to accommodate the required number of moves with the sliding window to determine support.

Given the branching nodes, all the possible path combinations are tested and if at least one has a sufficient number of $k_{rresolver}$ k-mers, the initially considered path of three nodes is considered supported. For example, in Additional file 1: Fig. S2, if the path in question is ARX, all the nodes preceding node A and succeeding node X that are within the sliding window moving distance would be used to form the path combinations to test. If ARX is a correct path, then at least one combination path should have reads, and so if any of them are found to be supported, then ARX is considered supported.

If the number of combinations explodes beyond a set threshold, the paths are randomly subsampled in order to limit run time and false positives. Bloom filter FPR is a factor in determining this threshold because increasing the number of tested paths increases the probability that a path will be supported by a series of false positive hits.

#### Repeat resolution

After tallying the found k-mers, the resulting supported paths might not unambiguously resolve paths in a repeat, but often simplify a repetitive region. Additional file 1: Fig. S3 shows an example of a possible simplification from the repeat in Additional file 1: Fig. S1. Despite not resolving all paths, a simplified repeat helps the downstream algorithms such as the contig and scaffolding stages of ABySS. In cases where paths are unambiguously resolved, nodes are immediately merged. The repeat simplification procedure is further explained in the Supplementary Repeat resolution section.

A summary flowchart of the algorithm can be seen in Additional file 1: Fig. S4. If the dataset used has multiple read sizes, the whole procedure is repeated for each size, starting from the shortest. Each read size works with a distinct $k_{rresolver}$ value either provided or automatically calculated.

### Performance assessment

RResolver is integrated in the ABySS 2 assembler [5] and works on the output of the DBG construction stage. Additional file 1: Fig. S5 shows how the method fits within the whole pipeline.

To assess the performance of RResolver and explore the parameter space, the method was tested on $2 \times 151$ bp and $2 \times 250$ bp Illumina data from four human individuals with fold-coverages ranging between 43$\times$ and 58$\times$. Additionally, the method was tested on $2 \times 110$ bp *C. elegans* and $2 \times 151$ bp *A. thaliana* datasets with 75$\times$ and 50$\times$ fold-coverages respectively. Finally, since RResolver improves ABySS assemblies, the performance of ABySS assembler with RResolver was benchmarked against other state-of-the-art short-read *de novo* assemblers. For these benchmarks, as well as using the four human datasets, the performance was assessed on the small *E. coli* genome, using $2$ bp, $2$ bp, and $2$ bp *E. coli* datasets of 209$\times$, 100$\times$, and 132$\times$ fold-coverages respectively. Assembly quality was assessed using QUAST [22] NGA50 and misassemblies, and BUSCO [23] gene completeness metrics. Further dataset information can be found in Methods.

**Fig. 1:**
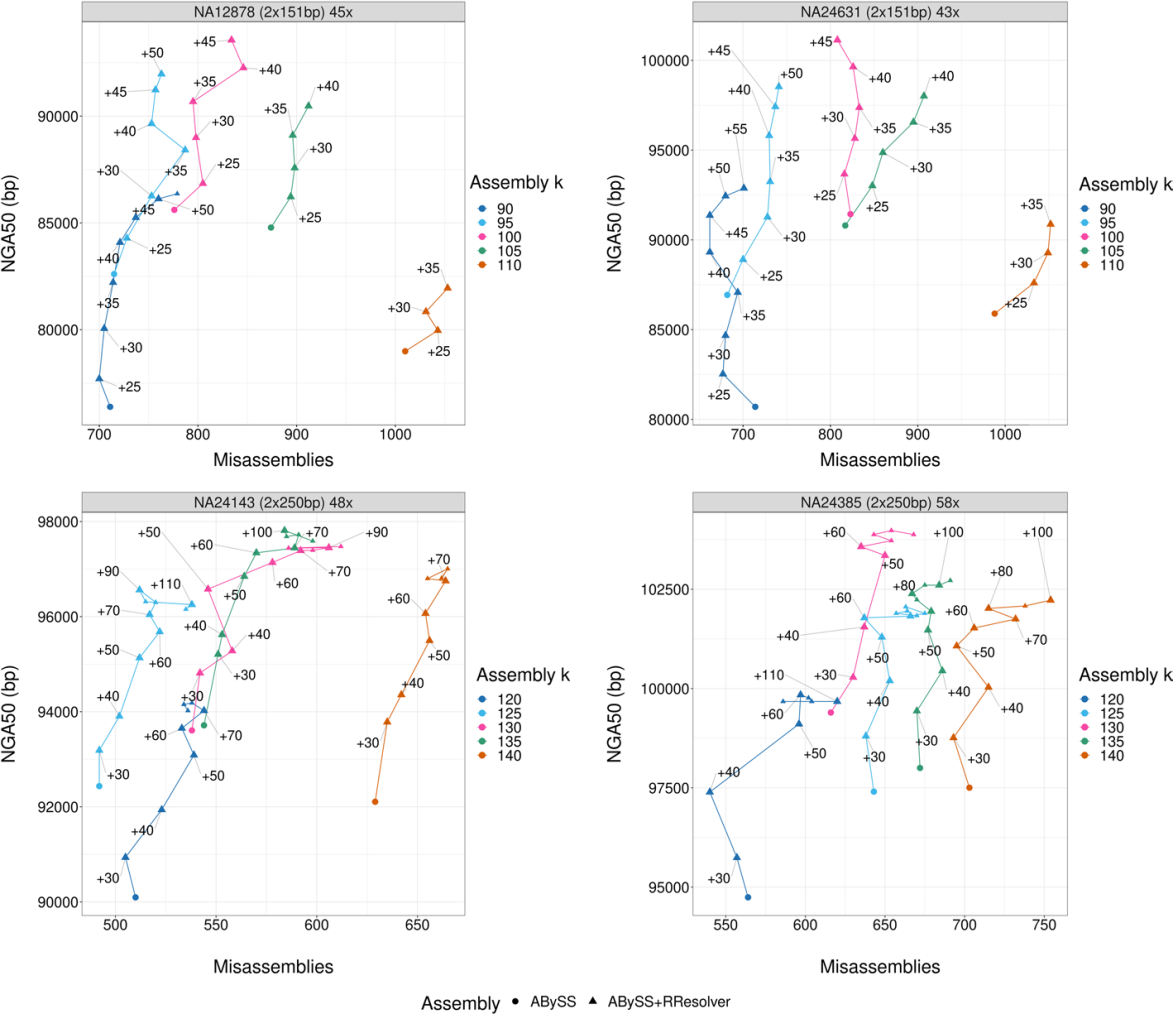
*H. sapiens* parameter sweep QUAST results. NGA50 and misassembly scaffold metrics with and without RResolver. High-quality assemblies lean towards top left corner, with high contiguity and low misassemblies. The text labels indicate the offset between $k_{rresolver}$ and $k_{assembly}$ used for each data point. Some text labels (for smaller triangles) and overlapping data points are omitted to reduce crowdedness in the plot, while keeping the trends. All RResolver data points have higher NGA50 than the corresponding baseline assembly, and some have fewer misassemblies. For $2$ bp datasets, picking the highest $k_{rresolver}$ increases NGA50 the most while keeping misassembly increase moderate. For $2$ datasets, picking the highest $k_{rresolver}$ is not necessarily optimal as it leads to increased misassemblies, and a $k_{rresolver} = k_{assembly} + 60$ is a good empirical choice for balancing NGA50 increase and minimizing misassemblies

Figure 1 and Additional file 1: Fig. S6 show ABySS *H. sapiens* assembly quality results (QUAST and BUSCO metrics respectively) for a range of $k_{assembly}$ and $k_{rresolver}$ sizes, with and without RResolver in the pipeline. For each dataset, a sweep with a step size of 5 bp on $k_{assembly}$ values was done in order to find the ABySS assembly without RResolver with the highest N50, as reported by the *abyss-fac* utility of the ABySS assembler. This is commonly done with ABySS assemblies in order to pick the optimal k-mer size. The highest N50 assembly was kept plus the ones with neighbouring $k_{assembly}$ values ($\pm 5$ and $\pm 10$). The choice of the $-kc$ ABySS parameter, which specifies the minimum k-mer multiplicity to filter out erroneous k-mers was also selected with the highest N50. The assemblies without RResolver are used as baseline upon which RResolver with various $k_{rresolver}$ values was tested.

Using the RResolver method, all ABySS human assemblies achieved higher NGA50 lengths (depending on the $k_{rresolver}$ value used, between 0.5% 15.1% relative increase) and most have higher percentage of complete BUSCO genes (up to 2.7% relative increase), while some have fewer misassemblies (up to 7.3% relative decrease and up to 13.7% relative increase). We explored the whole range of $k_{rresolver}$ values between $k_{assembly}$ and read size with a step size of 5 bp in order to assess the impact of the $k_{rresolver}$ parameter on assembly quality. This information was used to develop a heuristic for choosing $k_{rresolver}$ that would maximize contiguity and complete BUSCO genes and minimize misasssemblies in the absence of a reference.

For the $2 \times 151$ bp human reads, increasing $k_{rresolver}$ monotonically improves the NGA50 length and complete BUSCO genes for both datasets with a trend of somewhat increased misassemblies. Since RResolver does not make any cuts in the sequences, misassembly reduction found in some assemblies comes from repeat resolution enabling the downstream ABySS algorithms to more easily avoid making erroneous joins. Using the highest $k_{rresolver}$ value yields between 3.7 and 15.1% NGA50 relative increase, between 1.8% decrease and 11.0% increase of misassemblies, and between 0.5 and 2.7% complete BUSCO increase.

For the $2 \times 250$ bp human reads, increasing $k_{rresolver}$ as high as the read length can deteriorate assembly quality, as shown by increased misassemblies and diminishing trend of complete BUSCO genes. A difference of $+$ 60 between $k_{rresolver}$ and $k_{assembly}$ values on average yields increased NGA50 and increased complete BUSCO genes without too many additional misassemblies. Using the $k_{rresolver} = k_{assembly} + 60$ heuristic yields between 1.1 to 5.4% NGA50 relative increase, between 0.9% decrease and 7.4% increase of misassemblies, and between 0.25 and 0.8% complete BUSCO increase. For both read $2 \times 151$ bp and $2 \times 250$ bp, lower $k_{assembly}$ values benefit more, reducing the effect of a suboptimal $k_{assembly}$ value for the baseline assembly.

One of the reasons for limiting how high $k_{rresolver}$ value should go is the short-read base quality trend, which tends to drop sharply towards the read’s 3’ end [24]. This can be seen in the output of FastQC [25] for NA24631 ($2 \times 151$ bp) and NA24143 ($2 \times 250$ bp) in Additional file 1: Fig. S7. For $2 \times 151$ bp reads, Phred quality [26] starts noticeably dropping in the 140–150 bp range, whereas for 250 bp that happens in the 170–250 bp range.

**Fig. 2:**
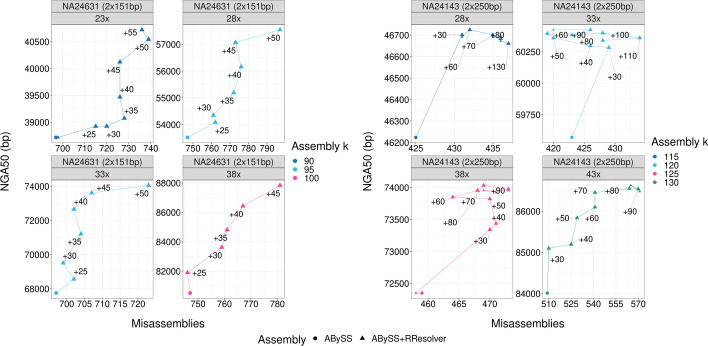
*H. sapiens* subsampled coverage QUAST results. NGA50 and misassemblies plots for a $2 \times 51$ bp and a $2 \times 250$ bp human dataset. The text labels indicate the offset between $k_{rresolver}$ and $k_{assembly}$ used for each data point. Each subplot ABySS base assembly uses optimal $k_{assembly}$ value. As in Fig. 1, the highest $k_{rresolver}$ is a good choice for $2 \times 151$ bp datasets, and an offset of $+$ 60 works well for $2 \times 250$ bp, giving a good contiguity improvement without increasing misassemblies too much

The results shown so far are for fold-coverages in the 40–60$\times$ range. Figure 2 shows the NGA50 and misassemblies metrics for a $2 \times 151$ bp and a $2 \times 250$ bp dataset with the read coverage subsampled down to 28$\times$ and 33$\times$ respectively with a step of 5$\times$ using seqtk [27]. The baseline ABySS assembly for each subplot uses the optimal $k_{assembly}$ value. Across all assemblies, for $2 \times 250$ bp reads, an offset of $+$ 60 between $k_{rresolver}$ and $k_{assembly}$ provides a balanced NGA50 increase while not introducing too many misassemblies. For $2 \times 151$ bp, the $+$ 60 offset is limited by read size, and so the highest $k_{rresolver}$ can be used as the optimal value. This confirms the heuristic of setting the $k_{rresolver}$ value to be 60 bp higher than $k_{assembly}$ and is the recommended approach if comparing assemblies that use different $k_{rresolver}$ values is too computationally costly or the reference is unavailable.

**Fig. 3:**
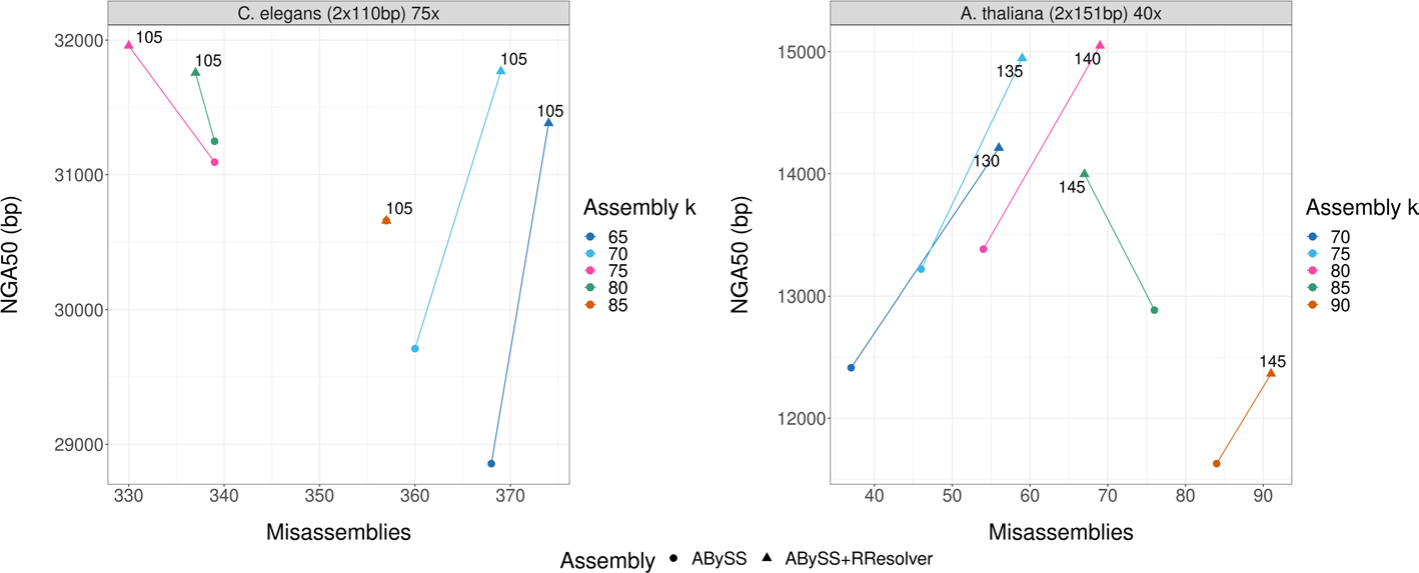
*C. elegans* and *A. thaliana* QUAST results. NGA50 and misassembly plots for *C. elegans* and *A. thaliana*. The $k_{rresolver} = k_{assembly} + 60\,\text{bp}$ heuristic is used, limited by read size of 110 bp and 151 bp. Both datasets see an improvement in contiguity, with a moderate increase in misassemblies in some cases. For C. elegans $k_{assembly} = 85$, no resolveable repeats were found and hence no change in assembly quality

To demonstrate that the algorithm performs well on genomes other than *H. sapiens* (3.1 Gbp haploid genome size), Fig. 3 and Additional file 1: Fig. S8 show results for $2 \times 110$ bp *C. elegans* and $2 \times 151$ bp *A. thaliana* datasets (101 Mbp and 157 Mbp genome sizes respectively). For both datasets, we applied the heuristic $k_{rresolver} = k_{assembly} + 60$ bp, with read size as the upper limit. For *C. elegans*, the baseline ABySS assembly with the highest NGA50 contiguity ($k_{assembly} = 80$ bp) does not yield the highest NGA50 contiguity final assembly with ABySS $+$ RResolver ($k_{assembly} = 75~\hbox {bp}, k_{rresolver} = 105~\hbox {bp}$). The assembly yielding the highest contiguity is the one with a lower $k_{assembly}$ which retains more connections in the graph. While this also results in more false edges, those can be removed by RResolver whereas it cannot recover connectivity lost by higher values of $k_{assembly}$. For *A. thaliana*, while all assemblies have increased contiguity (between 6.3 and 14.5% relative increase) and BUSCO completeness (between 0.1 and 0.3% relative increase), they come with a trend of increased misassemblies (between 20.9 and 51.4% relative increase). While the misassembly increase looks significant, there is a fairly low number of misassemblies in the first place and the absolute increase is not large (up to 19). In the case of *C. elegans*, contiguity has a relative increase between 0 and 8.8%, BUSCO completeness between 0 and 0.8%, and misassemblies have a relative increase of up to 2.5% and a decrease of up to 2.6%.

Along with the improved assembly quality, the average RResolver run time across all datasets when using the $k_{rresolver} = k_{assembly} + 60$ heuristic was only 2.4% of the whole ABySS pipeline, with the longest run time reaching 5.8%. For *H. sapiens* runs, with the heuristic the RResolver step took between 16 and 52 min, with 26 min being the average. Its memory usage for the same runs ranged between 54 and 63 GiB, with 57 GiB being the average. For all datasets, when using the heuristic the overall ABySS pipeline time increased on average by 0.5% compared to the baseline ABySS. However, pipeline peak memory usage increased on average by 8.4%. The machine specifications used for benchmarking can be found in Additional file 1: Table S1.

We also compared the ABySS assembler with RResolver to other state-of-the-art *de novo* assemblers to ensure its competitiveness. Additional file 1: Fig. S9 shows assembly quality results for the four human individuals with ABySS, DISCOVAR de novo [10], and MEGAHIT [21] assemblers. For the assemblies using $2 \times 151$ bp reads, ABySS produces the highest NGA50 length (93 Kbp and 101 Kbp) and BUSCO completeness (79.9% and 81.3%), and a comparable number of misassemblies (834 and 808) to DISCOVAR de novo (597 and 576). When comparing the assemblies using $2 \times 250$ bp reads, DISCOVAR de novo generates assemblies with higher contiguity (189 Kbp and 194 Kbp) and BUSCO completeness (86.0% and 86.1%), but a comparable number of misassemblies (295 and 340) to ABySS (578 and 635). For all datasets, MEGAHIT performs the worst in all three metrics. Additional file 1: Fig. S10 shows the run time and memory benchmarks for each tool. In terms of peak memory usage, DISCOVAR de novo is by far the most demanding, using 1.6–1.7 TiB for $2 \times 151$ bp datasets, and around 2TiB for $2 \times 250$ bp. ABySS memory usage peaks between 50 and 60 GiB, whereas MEGAHIT memory usage peaks between 80 and 120 GiB for all datasets. DISCOVAR de novo has the fastest run time, averaging 9 to 10 h per run. This is followed by ABySS which completes under a day for both $2 \times 151$ bp datasets and one $2 \times 250$ bp dataset, and a day and a half for the other $2 \times 250$ bp dataset. MEGAHIT has comparable run time to ABySS for $2 \times 151$ bp datasets, but runs over 2 days for $2 \times 250$ bp datasets.

When assembling small genomes such as *E. coli*, ABySS is comparable to SPAdes [8] and Unicycler [28] in all three metrics—NGA50 length, number of misassemblies, and BUSCO completeness. Additional file 1: Figs. S11 and S12 show assembly quality and resource usage comparison for $2 \times 100$ bp, $2 \times 150$ bp, and $2 \times 151$ bp *E. coli* datasets. All three assemblers have similar NGA50 contiguity (different at most by 2.6%) and misassembly count (differing at most by 43 misassemblies), with ABySS having more misassemblies on average. All assemblies recover 100% of the BUSCO complete genes. In terms of peak memory usage, ABySS uses less RAM by far ($<\hbox {5GiB}$, as opposed to 10–30 GiB for SPAdes and Unicycler) and has comparable run time to SPAdes with less than 15 min. Unicycler is the slowest, running on average between 30 and 60 min.

## Discussion

Resolving repeats in assembly graphs has been a widely researched topic. For DBGs, one way in which this has been achieved is using multiple k-mer sizes. The smaller sizes ensure connectivity in the graph whereas the larger sizes resolve repeats and untangle the graph. The current state-of-the-art methods have used multiple k-mer sizes, but only for smaller genomes, leaving a gap in the methodology. The studies so far have not addressed the scalability issues of their methods when dealing with large genomes. The concept of a multisized DBG, as used in the SPAdes assembler, relies on using multiple *k* values (i.e., k-mer sizes) to build the graph. This requires constructing contigs for each *k* value, which can be prohibitively slow for large genomes. Another approach, as employed by the IDBA assembler, is to make small *k* increments, making the exploration of a larger range of *k* values costly.

There are a number of challenges that come with attempting to use a multiple *k* values approach scalably—high memory usage, long execution times, complex repeats with a large number of possible paths, and errors. The work presented here addresses these challenges and the gap in the methodology, expanding upon the ways in which short-read information can be used to the fullest extent. In addition to the *k* value used by DBG, RResolver uses only one additional, larger *k* value in order to resolve repeats. This is different from the previous approaches of processing a list of *k* values and is a key enabler of scalability of the algorithm.

The two main aspects in which the RResolver algorithm could be improved are the $k_{rresolver}$ k-mer coverage estimation and handling of read errors. Coverage approximation is coarse, as the information available is at the contig level and so a higher resolution approximation could potentially help avoid erroneous resolutions. Read errors play a part in both coverage estimation, as they confound the number of reads that have contributed to a contig, and in missing $k_{rresolver}$ k-mers on queries, possibly resulting in mistakes.

If a read has an erroneous base call within the extracted $k_{rresolver}$ k-mers, RResolver will miss those k-mers when querying a correct path, reducing its support and potentially resulting in a misassembly. This is especially problematic for longer reads and larger $k_{rresolver}$ values, as there would be more bases that could be erroneous. It may be the case that the erroneous base call is found at the end of a $k_{rresolver}$ k-mer, while a preceeding $k_{assembly}$ k-mer of the same read might not have that error. This results in an inaccuracy in the proportion calculation in the coverage formula.

Another source of error is graph node read coverage. Since the ABySS assembler provides average $k_{assembly}$ k-mer multiplicity along a node, the information granularity decreases the longer the node is. For a particularly long node with highly varying coverage, this will lead to overestimation in low-coverage regions and to underestimation in high-coverage regions. Overestimating the number of expected $k_{rresolver}$ k-mers results in fewer tests done and therefore a greater chance of missing k-mers on a correct path. While it is possible to simply increase the number of tests overall by a factor, doing so reduces the number of repeats that can possibly be resolved, as the sliding window might not be long enough to do the required number of tests.

## Conclusions

Generating high-quality *de novo* assemblies is crucial for many downstream analyses. More contiguous and correct assemblies can greatly benefit various clinical applications and have found use in oncological projects [4]. Gene annotation can only go so far if the draft assembly being annotated is of limited quality [29], further emphasizing the point. However, improving *de novo* genome assemblies still has ways to go, as sequencing errors and repetitive sequences are major obstacles to achieving accurate assemblies [30].

In this work, we have demonstrated a method for improving the quality of *de novo* genome assemblies from short reads by utilizing unused range information. The presented algorithm, RResolver, resolves repeats in a DBG by storing large k-mers in a Bloom filter to estimate graph path support and remove unsupported paths. We have shown that the method consistently increases the contiguity of the assemblies and recovers fragmented or missing genes.

RResolver works seamlessly with the ABySS assembler pipeline, without requiring user involvement. When enabled, the output assembly benefits from higher quality. In this work, RResolver was tested on *H. sapiens*, *C. elegans*, *A. thaliana*, and *E. coli* genomes to assess performance on different genome sizes and complexities. Its execution time is only a fraction of the ABySS assembler pipeline it is a part of. We reported that on average RResolver increases the ABySS pipeline total run time by 2% and peak memory usage by 8%. The ABySS assembler was designed to work on large genomes, and so working within similar run time and memory constraints is important.

RResolver adds one more piece of the puzzle to generating high-quality *de novo* assemblies of large genomes and does so at the early stages of the assembly, benefiting any downstream algorithms that build contigs, scaffold the assembly, or do a final polishing.

## Methods

The RResolver algorithm runs the following steps in order:

- For each read size, starting from the shortest:
  1. Populate a Bloom filter with $k_{rresolver}$ k-mers.
  2. Identify repeats small enough to be spanned by $k_{rresolver}$ k-mers.
  3. Slide with a step of 1 bp a $k_{rresolver}$ sized window along all paths going through the identified repeats and query the Bloom filter on each step for presence of a k-mer.
  4. Delineate true from false paths using a threshold for the number of $k_{rresolver}$ k-mers found along each tested path.
  5. Modify the assembly graph to remove the false paths and leave the true paths. If this modification results in unambiguous paths of nodes, merge the nodes together.

The algorithm is summarized in the flowchart in Additional file 1: Fig. S4.

We used nine datasets in our experiments, assessing performance for different genome lengths and complexities, and for evaluating proper parameter choice. We used a *C. elegans* N2 strain dataset, with $2 \times 110$ bp paired end Illumina reads with 75-fold coverage. *A. thaliana* $2 \times 151$ bp paired end Illumina reads with 50-fold coverage. Four *H. sapiens* datasets — two $2 \times 151$ bp Illumina (NA12878, NA24631), and two $2 \times 250$ bp Illumina (NA24143, NA24385) paired end datasets were used with 45-, 43-, 48-, and 58-fold coverage respectively. The *H. sapiens* reference used for reference-based assessment was GRCh38. We also used three *E. coli* datasets with $2 \times 100$ bp, $2 \times 150$ bp, and $2 \times 151$ bp paired end Illumina reads with 209-, 100-, and 99-fold coverage respectively.

SRA and ENA accession IDs and links to download the data are provided in the Availability of data and materials section.

Assemblies were performed using ABySS v2.3.4, MEGAHIT v1.2.9, DISCOVAR de novo 52488, SPAdes v3.15.3, and Unicycler v0.4.8. ABySS without RResolver assemblies use ABySS v2.3.4 with RResolver disabled in the pipeline. For assembly evaluation, QUAST v5.0.2 and BUSCO v5.2.2 were used. Parameters used for each tool can be found in Additional file 1: Table S3.

In order to make k-mer extraction from reads and paths fast, ntHash [31], a rolling hash algorithm for nucleotide sequences, is used to efficiently calculate hashes of successive k-mers.

To consider a repeat for path evaluation, its length must be:

$$\begin{aligned} L_{repeat} \le k_{rresolver} - (tests - 1) - 2 \cdot margin \end{aligned}$$

where $L_{repeat}$ is the repeat length, *tests* the required number of tests (sliding window moves), and *margin* the minimum number of bases the sliding window should overlap on adjacent nodes at a minimum (2 by default).

The formula for the number of required tests is:

1 $$\begin{aligned} tests = max(m, s \cdot f + t) \end{aligned}$$

where *m* is the minimum number of tests (18), *s* the approximate space between neighbouring reads along the tested path, calculated by the coverage estimation formula described further in the text, *f* inaccuracy correction factor (4), and *t* support threshold (4). The minimum number of tests is enforced in order to make sure a path is not found unsuported due to not making a sufficient number of tests. The inaccuracy correction factor compensates for the errors of coverage estimation. Each read provides a number of $k_{rresolver}$ k-mers equal to the support threshold and so a constant equal to the threshold (*t*) is added to the formula to ensure that all extracted $k_{rresolver}$ k-mers are found. The parameter *M* sets the maximum number of tests (40). If tests is calculated as above *M*, the repeat is skipped. The numbers shown in parentheses are the default values, and are tunable through runtime parameters. Parameter *s* is calculated as:

2 $$\begin{aligned} s = \frac{L - l + 1}{R_p} \end{aligned}$$

where *L* is the length of the tested path, *l* read length, and $R_p$ the number of reads that have contributed to the path during the assembly DBG stage. Calculating $R_p$ is further described in the Supplementary Varying coverage section. This number is approximated based on the $k_{assembly}$ coverage of the path, provided by the assembler.

Equation 2 is only an approximation and its output should be interpreted carefully. To make sure no reads are missed, in Eq. 1 its output is multiplied by a factor of 4. The same rationale is behind setting a minimum number of sliding window moves (18). The formula may overestimate the number of $k_{rresolver}$ k-mers expected and perform too few tests, which this lower limit prevents.

To consider a path supported, a threshold of 4 $k_{rresolver}$ k-mers is used. Additionally, 4 $k_{rresolver}$ k-mers are extracted per read, starting from the 5’ end, reducing the effect of the read quality drop towards the 3’ end [24]. The number of hash functions per $k_{rresolver}$ k-mers when inserting into the Bloom filter is 7.

Additional file 1: Fig. S13 shows the histogram of $k_{rresolver}$ k-mers found along all the tested paths for the $k_{assembly} = 100, k_{rresolver} = 145$ *H. sapiens* NA24631 assembly with a threshold of 4. There is a clear separation between the two distributions of unsupported and supported paths, with the first noticeable histogram bar of supported paths at 4 k-mers suggesting that the threshold of 4 is appropriate. The paths with Bloom filter false positives are found between the two distributions, however, due to low FPR of $7.57 \cdot 10^{-11}$ for this assembly, they are few and not visible. The spike at 18 k-mers is due to a default minimum number of sliding window moves of 18.

When dealing with complex repeats (Additional file 1: Fig. S2), a maximum of 75 paths are allowed on either side of the repeat for a maximum total of 5625 path combinations. In case there are more than maximum, the paths are randomly subsampled down to 5625.

Two iterations of graph path evaluation and resolution are done per read size, as the path evaluation completes very quickly and can uncover additional opportunities for repeat resolution.

## Additional file

**Additional file 1. **Supporting data (supplementary methods, figures and tables) for RResolver: efficient short-read repeat resolution within ABySS.

## Data Availability

The accession IDs and URLs to the datasets used in this study can be found in Additional file 1: Table S2. Source code can be downloaded from: https://github.com/bcgsc/abyss/tree/master/RResolver. The ABySS release with the RResolver algorithm used in the results can be downloaded from: https://github.com/bcgsc/abyss/releases/tag/2.3.4. ABySS README includes relevant tutorials and sample synthetic data.

## References

[1] Warren RL, Keeling CI, Yuen MMS, Raymond A, Taylor GA, Vandervalk BP, Mohamadi H, Paulino D, Chiu R, Jackman SD, Robertson G, Yang C, Boyle B, Hoffmann M, Weigel D, Nelson DR, Ritland C, Isabel N, Jaquish B, Yanchuk A, Bousquet J, Jones SJM, MacKay J, Birol I, Bohlmann J (2015). Improved white spruce (*Picea glauca*) genome assemblies and annotation of large gene families of conifer terpenoid and phenolic defense metabolism. Plant J.

[2] Fitz-Gibbon S, Hipp AL, Pham KK, Manos PS, Sork VL (2017). Phylogenomic inferences from reference-mapped and de novo assembled short-read sequence data using RADseq sequencing of california white oaks (quercus section quercus). Genome.

[3] Das P, Sahoo L, Das SP, Bit A, Joshi CG, Kushwaha B, Kumar D, Shah TM, Hinsu AT, Patel N, Patnaik S, Agarwal S, Pandey M, Srivastava S, Meher PK, Jayasankar P, Koringa PG, Nagpure NS, Kumar R, Singh M, Iquebal MA, Jaiswal S, Kumar N, Raza M, Mahapatra KD, Jena J (2020). De novo assembly and genome-wide SNP discovery in rohu carp, labeo rohita. Front Genet.

[4] Jamshidi F, Pleasance E, Li Y, Shen Y, Kasaian K, Corbett R, Eirew P, Lum A, Pandoh P, Zhao Y, Schein JE, Moore RA, Rassekh R, Huntsman DG, Knowling M, Lim H, Renouf DJ, Jones SJM, Marra MA, Nielsen TO, Laskin J, Yip S (2014). Diagnostic value of next-generation sequencing in an unusual sphenoid tumor. Oncologist.

[5] Jackman SD, Vandervalk BP, Mohamadi H, Chu J, Yeo S, Hammond SA, Jahesh G, Khan H, Coombe L, Warren RL, Birol I (2017). ABySS 2.0: resource-efficient assembly of large genomes using a bloom filter. Genome Res..

[6] Chikhi R, Rizk G (2013). Space-efficient and exact de Bruijn graph representation based on a bloom filter. Algorithms Mol Biol.

[7] Zerbino DR, Birney E (2008). Velvet: algorithms for de novo short read assembly using de Bruijn graphs. Genome Res.

[8] Bankevich A, Nurk S, Antipov D, Gurevich AA, Dvorkin M, Kulikov AS, Lesin VM, Nikolenko SI, Pham S, Prjibelski AD, Pyshkin AV, Sirotkin AV, Vyahhi N, Tesler G, Alekseyev MA, Pevzner PA (2012). SPAdes: a new genome assembly algorithm and its applications to single-cell sequencing. J Comput Biol.

[9] Luo R, Liu B, Xie Y, Li Z, Huang W, Yuan J, He G, Chen Y, Pan Q, Liu Y, Tang J, Wu G, Zhang H, Shi Y, Liu Y, Yu C, Wang B, Lu Y, Han C, Cheung DW, Yiu S-M, Peng S, Xiaoqian Z, Liu G, Liao X, Li Y, Yang H, Wang J, Lam T-W, Wang J (2012). SOAPdenovo2: an empirically improved memory-efficient short-read de novo assembler. GigaScience.

[10] DISCOVAR: Assemble genomes, find variants. https://www.broadinstitute.org/software/discovar/blog. Accessed 8 Apr 2020

[11] Simpson JT, Wong K, Jackman SD, Schein JE, Jones SJM, Birol I (2009). ABySS: a parallel assembler for short read sequence data. Genome Res.

[12] Bloom BH (1970). Space/time trade-offs in hash coding with allowable errors. Commun ACM.

[13] Vandervalk BP, Yang C, Xue Z, Raghavan K, Chu J, Mohamadi H, Jackman SD, Chiu R, Warren RL, Birol I (2015). Konnector v2.0: pseudo-long reads from paired-end sequencing data. BMC Med Genom.

[14] Warren RL, Yang C, Vandervalk BP, Behsaz B, Lagman A, Jones SJM, Birol I (2015). LINKS: scalable, alignment-free scaffolding of draft genomes with long reads. GigaScience.

[15] de Koning APJ, Gu W, Castoe TA, Batzer MA, Pollock DD (2011). Repetitive elements may comprise over two-thirds of the human genome. PLoS Genet.

[16] Kidwell MG (2002). Genetica.

[17] Chalopin D, Naville M, Plard F, Galiana D, Volff J-N (2015). Comparative analysis of transposable elements highlights mobilome diversity and evolution in vertebrates. Genome Biol Evol.

[18] Bansal V, Boucher C (2019). Sequencing technologies and analyses: where have we been and where are we going?. iScience.

[19] Ekblom R, Smeds L, Ellegren H (2014). Patterns of sequencing coverage bias revealed by ultra-deep sequencing of vertebrate mitochondria. BMC Genom.

[20] Peng Y, Leung HCM, Yiu SM, Chin FYL, Berger B (2010). Idba—a practical iterative de Bruijn graph de novo assembler. Research in Computational Molecular Biology.

[21] Li D, Liu C-M, Luo R, Sadakane K, Lam T-W (2015). MEGAHIT: an ultra-fast single-node solution for large and complex metagenomics assembly via succinct de Bruijn graph. Bioinformatics.

[22] Gurevich A, Saveliev V, Vyahhi N, Tesler G (2013). QUAST: quality assessment tool for genome assemblies. Bioinformatics.

[23] Simão FA, Waterhouse RM, Ioannidis P, Kriventseva EV, Zdobnov EM (2015). BUSCO: assessing genome assembly and annotation completeness with single-copy orthologs. Bioinformatics.

[24] Dohm JC, Lottaz C, Borodina T, Himmelbauer H (2008). Substantial biases in ultra-short read data sets from high-throughput DNA sequencing. Nucleic Acids Res.

[25] Andrews S, Krueger F, Segonds-Pichon A, Biggins L, Krueger C, Wingett S (2010). FastQC.

[26] Ewing B, Hillier L, Wendl MC, Green P (1998). Base-calling of automated sequencer traces UsingPhred. I. Accuracy assessment. Genome Res.

[27] Seqtk, a fast and lightweight tool for processing sequences in the FASTA or FASTQ format. https://github.com/lh3/seqtk. Accessed 13 Jan 2021.

[28] Wick RR, Judd LM, Gorrie CL, Holt KE (2017). Unicycler: resolving bacterial genome assemblies from short and long sequencing reads. PLoS Comput Biol.

[29] Salzberg SL (2019). Next-generation genome annotation: we still struggle to get it right. Genome Biol.

[30] Denton JF, Lugo-Martinez J, Tucker AE, Schrider DR, Warren WC, Hahn MW (2014). Extensive error in the number of genes inferred from draft genome assemblies. PLoS Comput Biol.

[31] Mohamadi H, Chu J, Vandervalk BP, Birol I (2016). ntHash: recursive nucleotide hashing. Bioinformatics.

