# Supplementary material for: RResolver: efficient short-read repeat resolution within ABySS
Source: BMC Bioinformatics. 2022 Jun 21;23:246. doi: 10.1186/s12859-022-04790-z (PMC9215042; doi:10.1186/s12859-022-04790-z)
Supplement: Supplementary file 1 — Additional file 1. Supporting data (supplementary methods, figures and tables) for RResolver: efficient short-read repeat resolution within ABySS. [file 12859_2022_4790_MOESM1_ESM.pdf]

Supplementary Data

Contents

Supplementary methods **2**
Varying coverage . . . 2
Repeat resolution . . . 2

Supplementary figures **3**

Supplementary tables **15**

Supplementary methods

Varying coverage

R_p, the number of reads that have contributed to a path in the de Bruijn Graph (DBG) graph is obtained as:

$$R_p = \frac{b_p \cdot R_{b\%}}{k_{assembly} \cdot (l - k_{assembly} + 1)} \tag{1}$$

where b_p is the sum of bases contributed to the path in question from reads of all sizes, $R_{b\%}$ the fraction of bases contributed by reads of currently processed read size out of bases from reads of all sizes. In this context, a k-mer of length k contributes k bases, and a read of length l contributes $k \cdot (l - k + 1)$ bases, as there are $(l - k + 1)$ k-mers in a read of length l. The ABySS assembler provides the number of $k_{assembly}$ k-mers that have contributed to each contig and from there we can obtain b_p.

$R_{b\%}$ is calculated as:

$$R_{b\%} = \frac{R_{\%} \cdot (l - k_{assembly} + 1)}{\sum_{i=1}^{n} R_{\%}^{i} \cdot (l^i - k_{assembly} + 1)} \tag{2}$$

Where $R_{\%}$ is the proportion of the currently processed read size in the input files. The sum in the denominator iterates over all read sizes, summing up each read size base contribution. For each read size i, $R_{\%}^{i}$ is approximately calculated by sampling the input reads.

Since the path consists of three nodes: the repeat node and the two adjacent nodes, we can use their k-mer coverage as the coverage of the path. Among the three nodes, the lowest coverage is taken to be the coverage of the path, as the higher coverage of other nodes can be explained by different paths going through them. $cov_{k_{assembly}}$ is provided by the ABySS assembler for every contig.

Repeat resolution

Once all considered paths are examined, for each repeat, a map is constructed that that has supported outgoing nodes for each incoming node. As this may not unambiguously resolve the paths, a simplification procedure is implemented. Incoming nodes are grouped based on which outgoing nodes they have correct paths to. All incoming nodes with the exact same set of outgoing nodes form one group. For example, if in Figure S1 the true paths are determined to be ARX, ARY, BRX, BRY, CRY, CRZ, incoming nodes A and B would be grouped together as they both go to X and Y, whereas node C would be in its own group as it goes to Y and Z (Figure S14). Each group then forms an instance of the repeat, resulting in all paths being supported in the new subgraph.

Supplementary figures

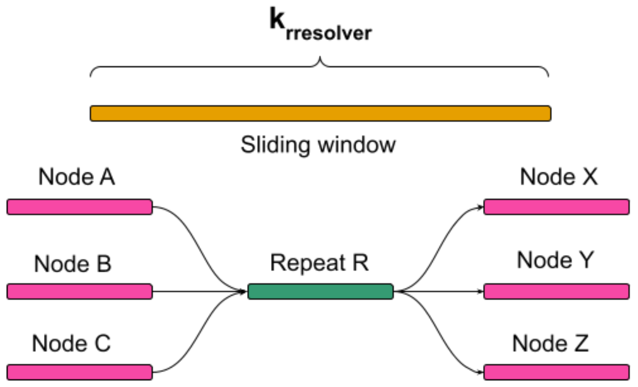

Figure S1: A resolveable repeat candidate. For a repeat to be considered, it has to be short enough so that the window of $k_{rresolver}$ size spans the repeat, the adjacent nodes, and has sufficient room to do a number of moves, i.e., tests, dynamically determined based on local sequencing coverage. All possible paths are tested for support: ARX, ARY, ARZ, BRX, BRY, BRZ, CRX, CRY, CRZ.

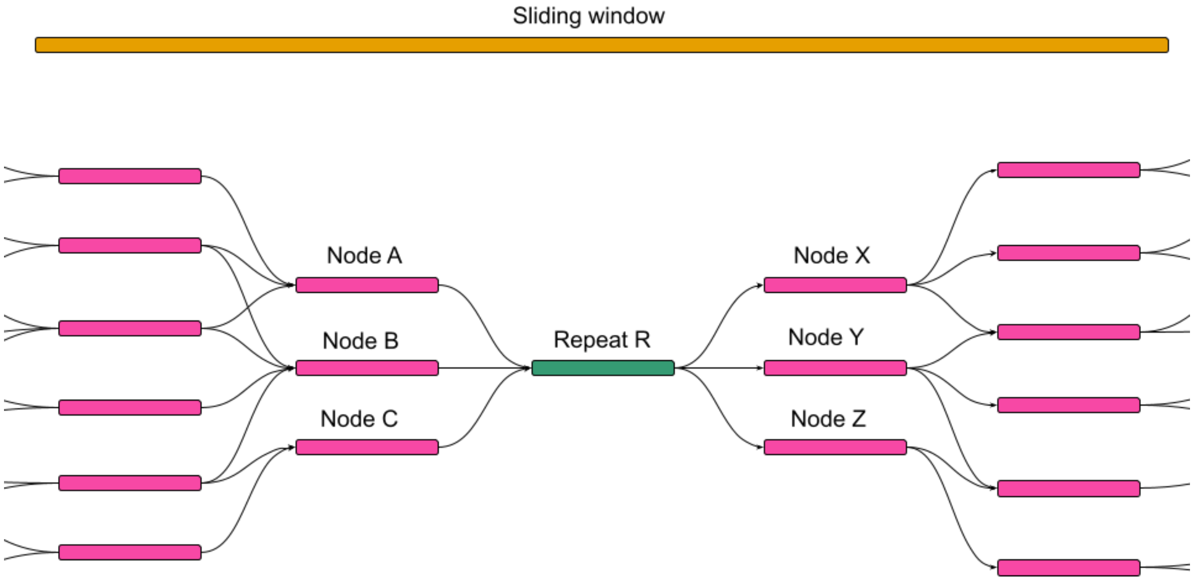

Figure S2: Branching in a complex repeat. In complex repeats with a large number of paths, the nodes adjacent to the tested repeat tend to have short sequences before branching further. If the sequence from the path being tested is shorter than the window, the only way to test the path is to add the sequences of the nodes further away. E.g. if the tested path is ARX, all combinations of paths from the nodes preceding node A to all the nodes succeeding node X that fit within the sliding window moving distance are considered. Support found in any of the paths is sufficient to consider the ARX path supported.

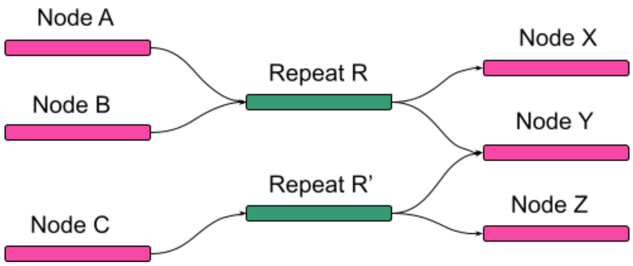

Figure S3: A simplified repeat. If the correct paths in Figure S1 were found to be ARX, ARY, BRX, BRY, CRY, CRZ, the subgraph would be simplified as shown in the figure, where R' is a copy of the original R node. Although the only nodes that can be unambiguously merged here are C and R', narrowing down possible paths benefits downstream algorithms.

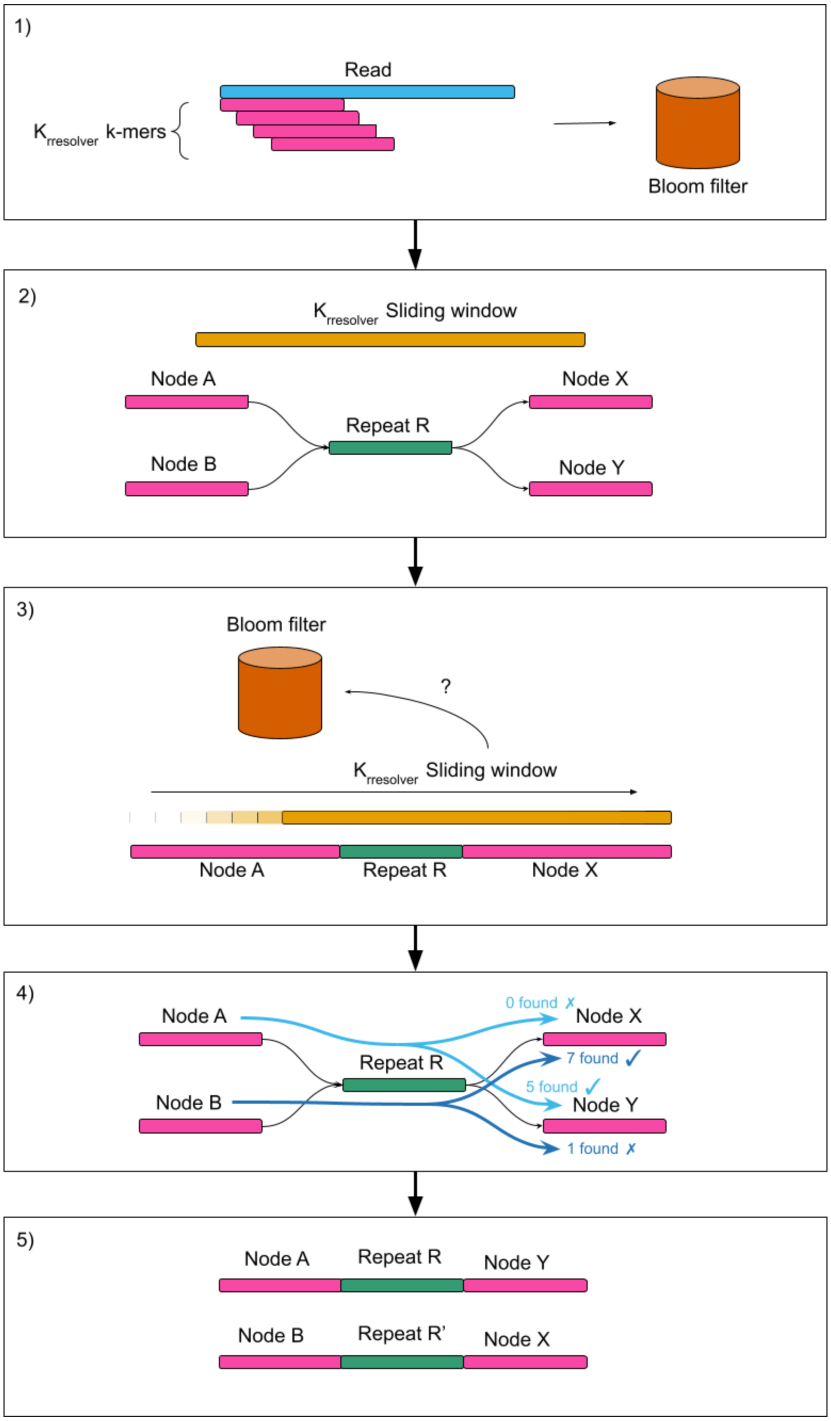

Figure S4: Flowchart of the RResolver algorithm. 1) $k_{rresolver}$ k-mers are extracted from input reads and inserted into a Bloom Filter. 2) Small repeats that can be spanned by the $k_{rresolver}$ window are identified. 3) All possible paths in the repeat are tested by sliding the window along them with 1bp slide step size, querying the Bloom filter on every step. 4) Paths with sufficient number of $k_{rresolver}$ k-mers are identified. 5) Repeats are simplified by removing unsupported paths and joining supported ones.

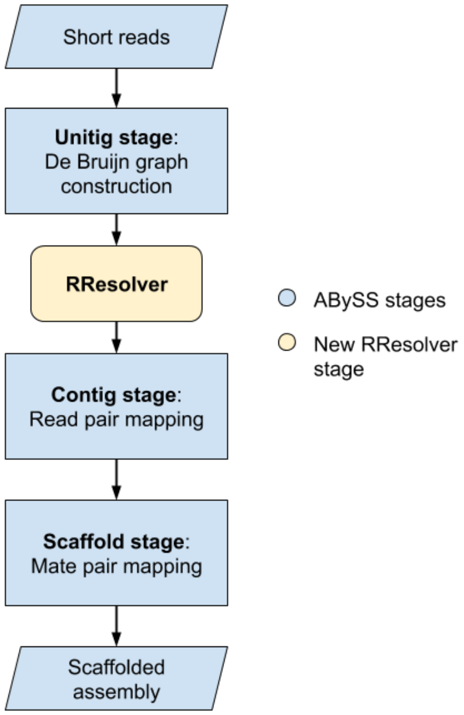

Figure S5: RResolver is ran after the DBG stage of the ABySS pipeline, benefiting the downstream contig and scaffold stage with an improved graph.

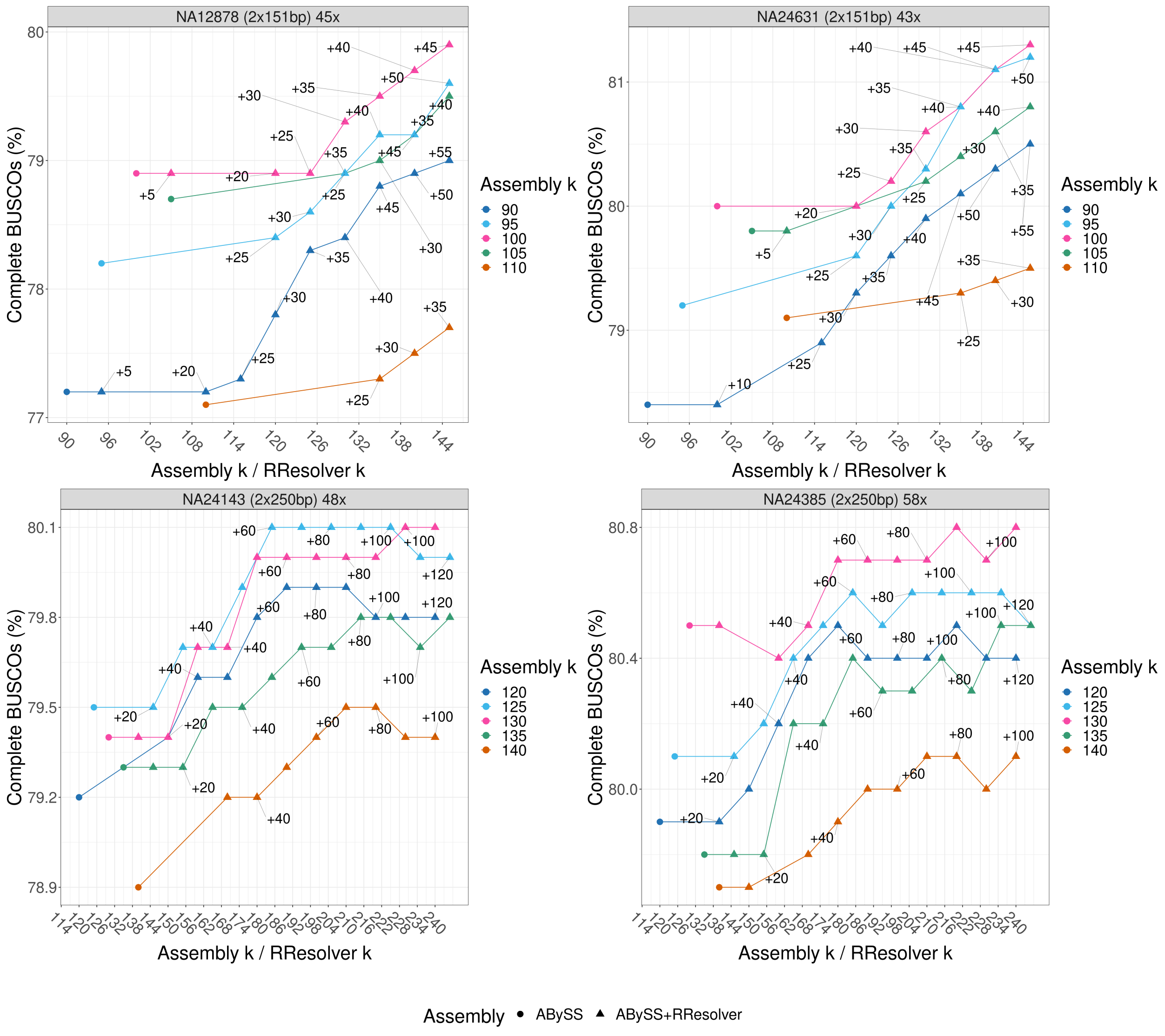

Figure S6: *H. sapiens* parameter sweep BUSCO results. Similar to Figure 1 in the main text, the plot shows an exploration of $k_{rresolver}$ values and its effect on complete BUSCO genes. Like in the QUAST results figure, 2x151bp datasetes benefit the most from the highest $k_{rresolver}$. Similarly, BUSCO completeness plateaus past $k_{rresolver} = k_{assembly} + 60$.

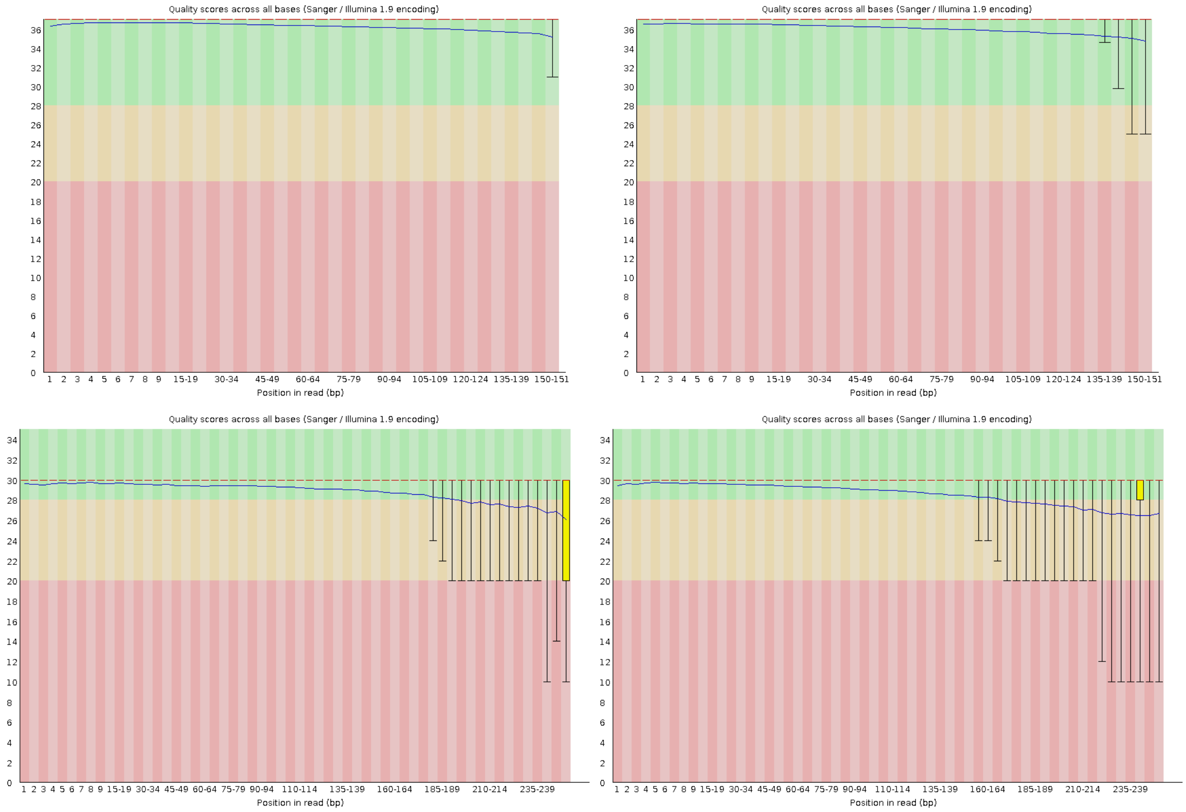

Figure S7: The output of FastQC for the *H. sapiens* NA24631 (2x151bp) and NA24143 (2x250bp) reads in first and second row respectively. The common pattern of base quality distribution can be seen — high quality bases from the start with a sharp drop at the end of the read.

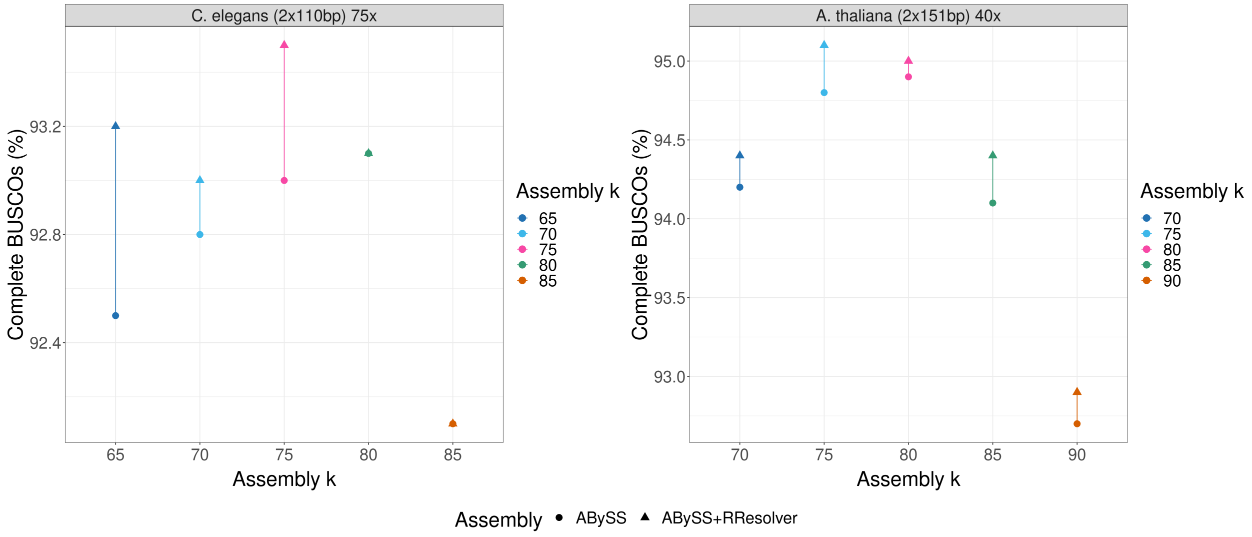

Figure S8: *C. elegans* and *A. thaliana* BUSCO results. Similar to the Figure 3 in the main text, instead showing BUSCO results. All *A. thaliana* ABySS+RResolver datapoints see an increase in BUSCO completness, while most *C. elegans* ABySS+RReslver datapoints have higher completeness, with the exception of the ones with higher $k_{assembly}$ values which had fewer repeat resolution opportunities.

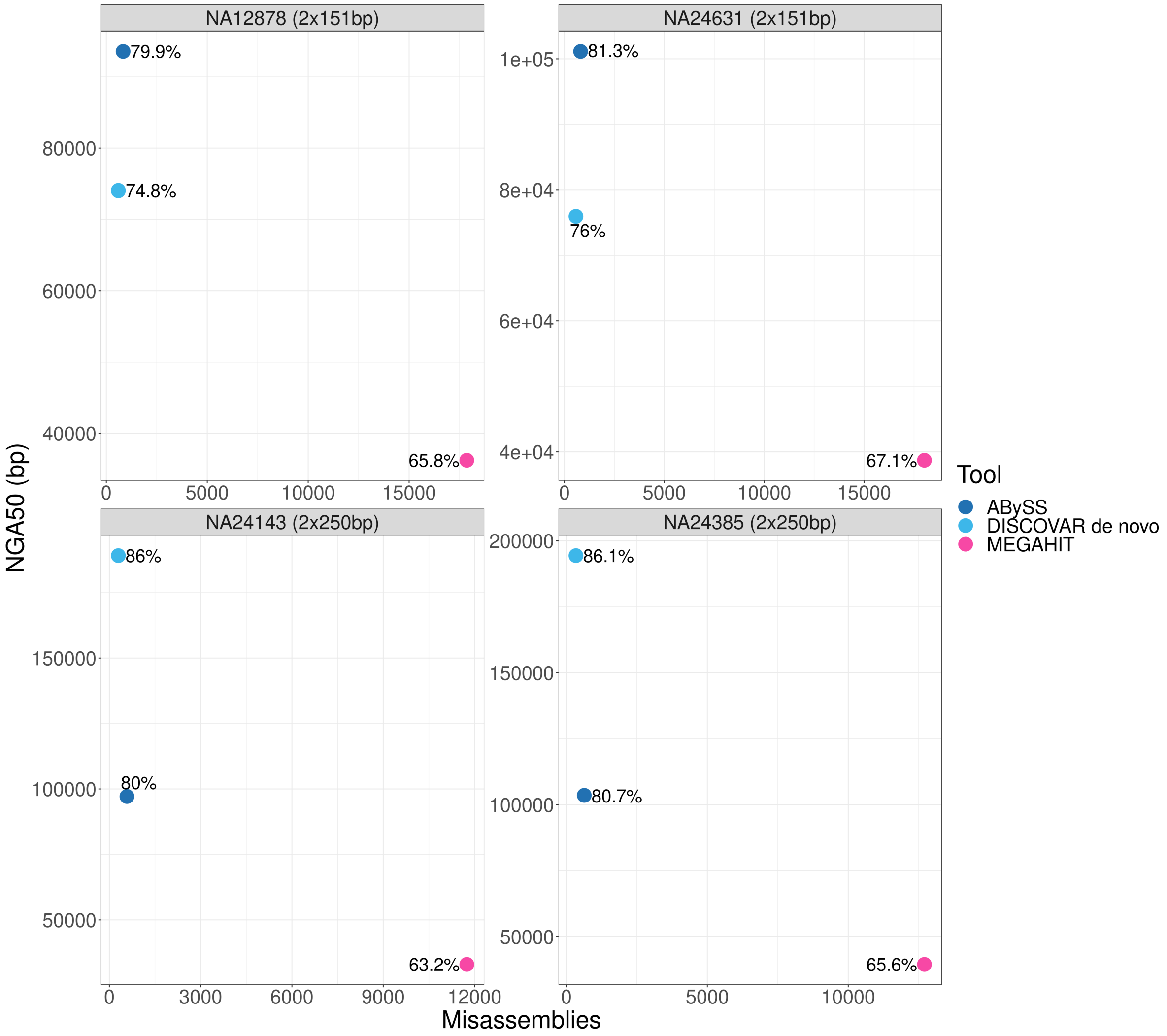

Figure S9: NGA50, misassemblies (axes), and BUSCO gene completeness (labels) as measured by QUAST and BUSCO for the four human datasets and the three compared assemblers: ABySS, DISCOVAR de novo, and MEGAHIT. For 2x151bp datasets, ABySS assemblies have the highest NGA50 contiguity and BUSCO completeness, and comparable misassemblies with DISCOVAR de novo. for 2x250bp datasets, DISCOVAR de novo assemblies have the highest NGA50 contiguity and BUSCO completeness and are comparable in the number of misassemblies to ABySS. For all datasets, MEGAHIT performs the worst by all three metrics.

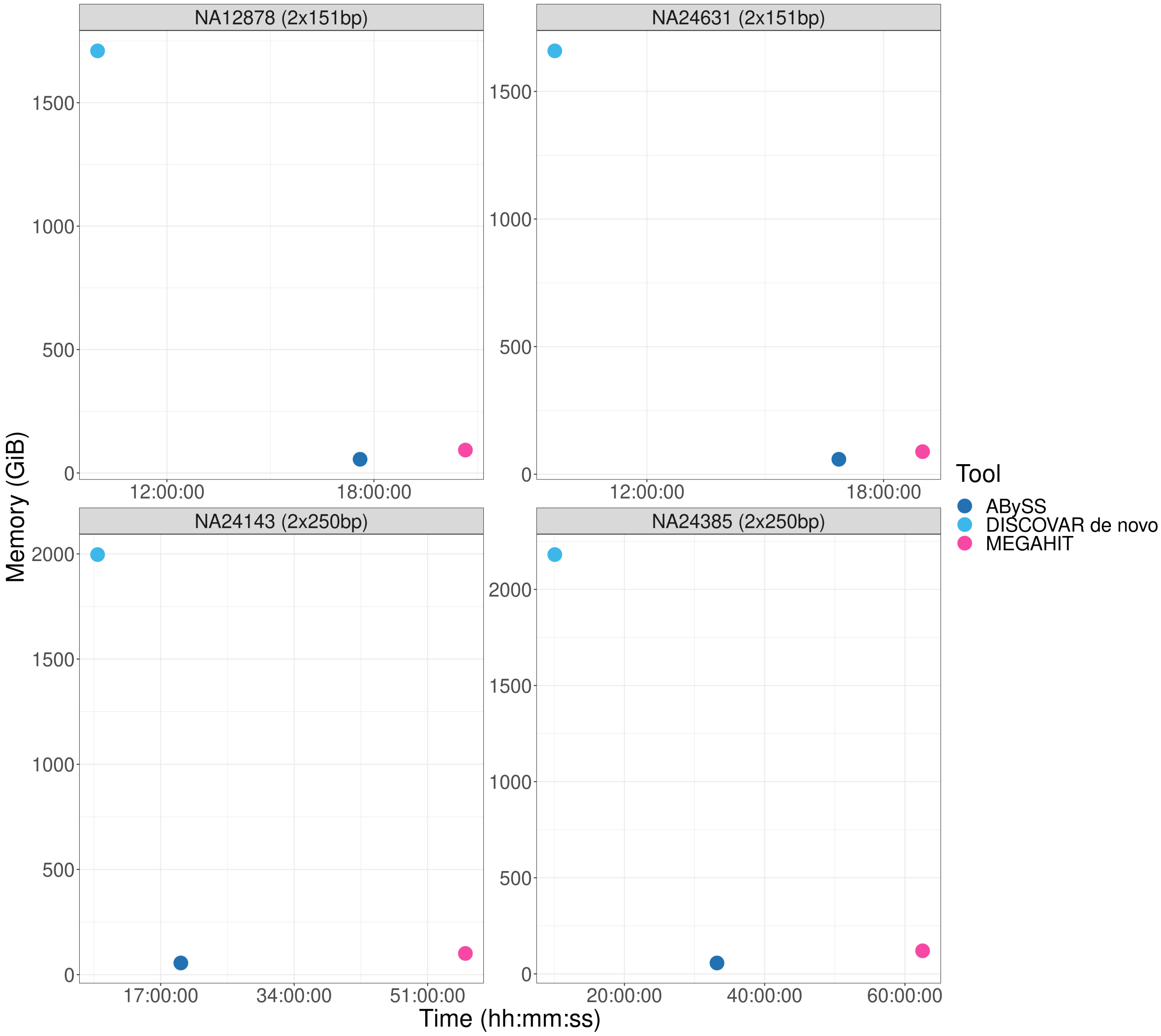

Figure S10: Memory usage and run time for the four human individuals and the three assemblers. DISCOVAR uses the most memory – around 1.6TiB for 2x151bp datasets, and around 2TiB for 2x250bp. For all datasets, ABySS and MEGAHIT have similar memory requirements, below 100GiB for ABySS and around 100GiB for MEGAHIT. DISCOVAR de novo has the best run time, averaging around 9 hours per run. ABySS runs under a day for both 2x151bp datasets and one 2x250bp dataset, and a day and a half for the other 2x250bp dataset. MEGAHIT has comparable run time for 2x151bp datasets, but runs over two days for 2x250bp datasets.

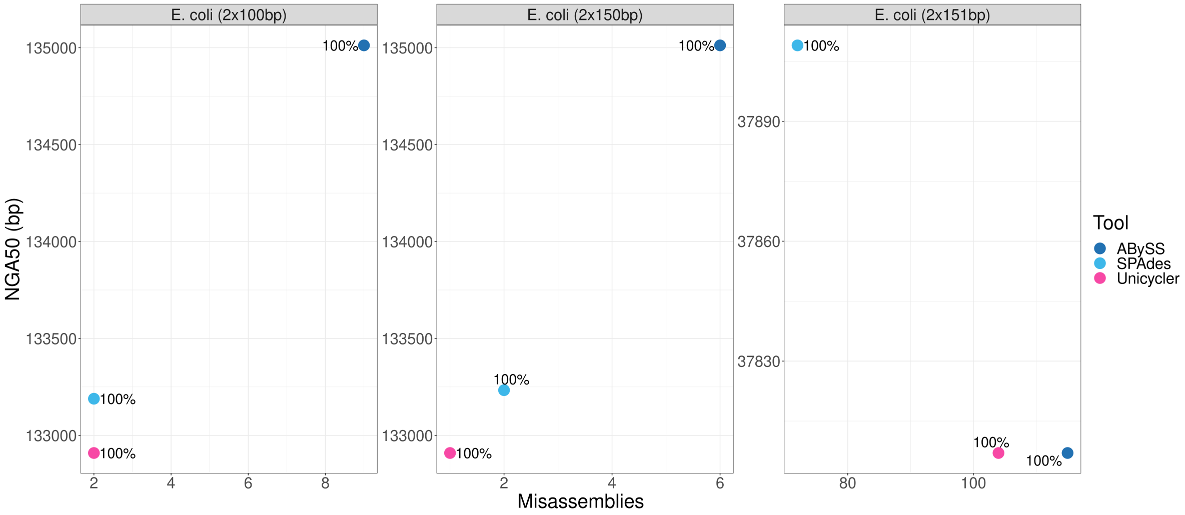

Figure S11: NGA50, misassemblies (axes), and BUSCO gene completeness (labels) as measured by QUAST and BUSCO for the three *E. coli* datasets. All three assemblers have comparable NGA50 contiguity and misassembly count, with ABySS having more misassemblies on average. All assemblies recover 100% of the BUSCO complete genes.

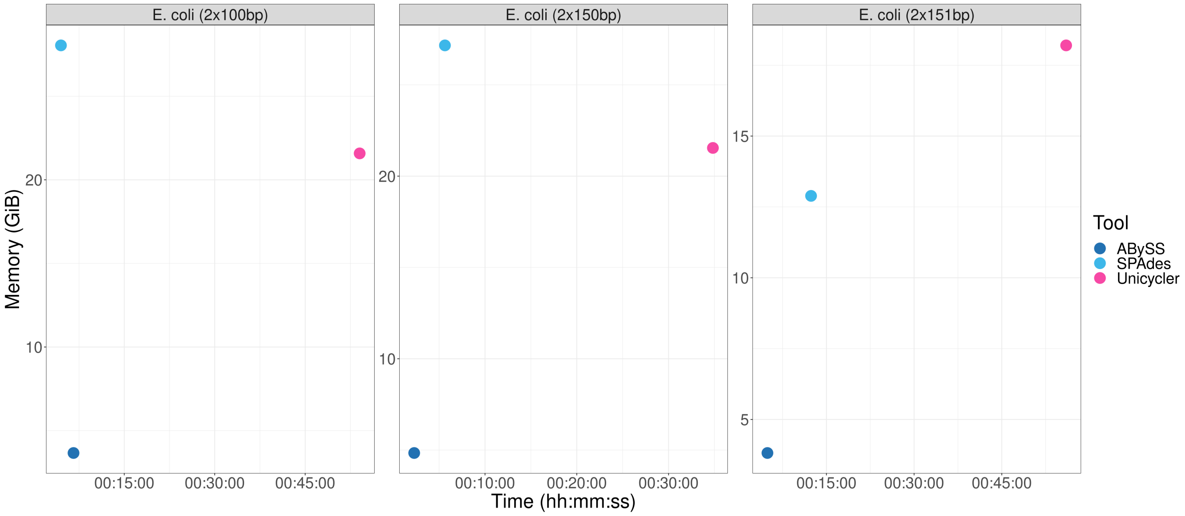

Figure S12: Run time and memory usage for the three *E. coli* datasets. ABySS uses the least by far and has comparable run time to SPAdes with around 15 minutes or less. Unicycler is the slowest, running on average between 30 and 60 minutes.

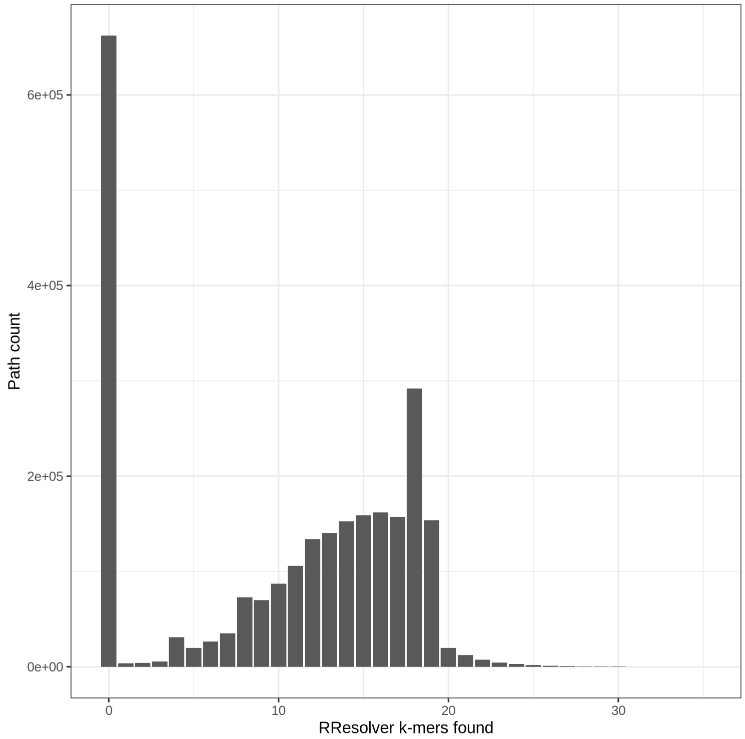

Figure S13: Histogram of the number of $k_{rresolver}$ k-mers found along all tested paths for *H. sapiens* NA24631 assembly ($k_{assembly} = 100, k_{rresolver} = 145$). The number of extracted k-mers per read is 4 and as can be seen on the figure, a threshold of 4 to consider a path supported separates the distributions of unsupported and supported paths well. The paths with Bloom filter false positives are found between the two distributions, but because of the low FPR, they are few and not visible. The spike at 18 k-mers found is due to the lower limit on the number of sliding window moves of 18.

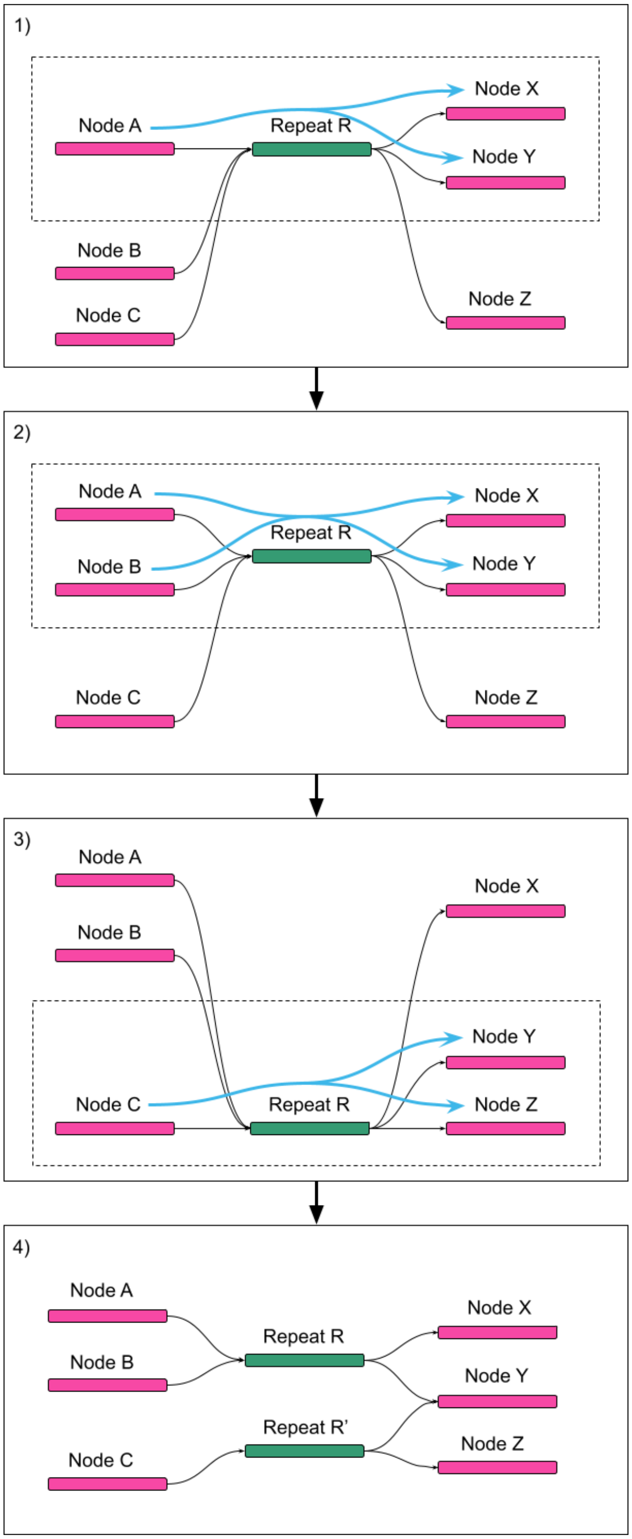

Figure S14: Step by step repeat simplification process. 1) Node A is identified to have correct paths to nodes X and Y. 2) Node B is also identified to have correct paths to X and Y, forming the same group as node A. 3) Node C has correct paths to different nodes, Y and Z, forming its own group. 4) Each group forms an instance of the repeat where all of the group's incoming nodes go to the same outgoing nodes.

Supplementary tables

Table S1: Machine specifications for run time and memory benchmarking.

Tool	CPU	RAM	OS
ABySS v2.3.4<br>MEGAHIT v1.2.9<br>SPAdes v3.15.3<br>Unicycler v0.4.8	48 x Intel Xeon E5-2650 @ 2.20GHz	377 GB	CentOS 7
DISCOVAR de novo 52488	144 x Intel Xeon Gold 6254 CPU @ 3.10GHz	3.0T	CentOS 7

Table S2: Data sources.

Data	Accession & URL
NA12878 2x151bp	ERR3685389<br>`https://www.ebi.ac.uk/ena/browser/view/ERR3685389`
NA24631 2x151bp	ERR3687419<br>`https://www.ebi.ac.uk/ena/browser/view/ERR3687419`
NA24385 2x250bp	SRR11321732<br>`https://trace.ncbi.nlm.nih.gov/Traces/sra/?run=SRR11321732`
NA24143 2x250bp	SRR11321730<br>`https://trace.ncbi.nlm.nih.gov/Traces/sra/?run=SRR11321730`
H. sapiens reference GRCh38	`ftp://ftp.ncbi.nlm.nih.gov/genomes/all/GCA/000/001/405/GCA_000001405.15_GRCh38/seqs_for_alignment_pipelines.ucsc_ids`
C. elegans 2x110bp	DRR008444<br>`https://www.ncbi.nlm.nih.gov/sra/?term=DRR008444`
C. elegans reference	`ftp://ftp.ncbi.nlm.nih.gov/genomes/archive/old_refseq/Caenorhabditis_elegans`
A. thaliana 2x151bp	SAMD00000601<br>`https://www.ebi.ac.uk/ena/browser/view/SAMD00000601`
A. thaliana reference	`https://www.ncbi.nlm.nih.gov/assembly/GCF_000001735.4`
E. coli 2x100bp (downsampled to 209x)	SRR13921546<br>`https://www.ncbi.nlm.nih.gov/sra/?term=SRR13921546`
E. coli 2x150bp (downsampled to 100x)	SRR11558946<br>`https://www.ncbi.nlm.nih.gov/sra/?term=SRR11558946`
E. coli 2x151bp	ERR3713237<br>`https://www.ebi.ac.uk/ena/browser/view/ERR3713237`
E. coli reference K12 (NC_000913.3)	`https://www.ncbi.nlm.nih.gov/nuccore/NC_000913.3`

Table S3: ABySS, MEGAHIT, DISCOVAR de novo, SPAdes, Unicycler, QUAST, and BUSCO parameters used to assemble and assess tested genomes. N/A denotes no parameters that affect the output were supplied. The $-k$ parameter for ABySS is omitted, as a sweep of values was done. BUSCO dataset creation dates for enterobacterales_odb10, nematoda_odb10, brassicales_odb10, and primates_odb10 are 2021-02-23, 2020-08-05, 2020-08-05, and 2021-02-19 respectively.

**Tool**	**Dataset**	**Parameters**
ABySS v2.3.4	*E. coli* 2x100bp<br>*E. coli* 2x150bp<br>*E. coli* 2x151bp	-kc2 -B2G -H4 -j48
	C. elegans 2x110bp	-kc2 -B5G -H4 -j48
	A. thaliana 2x151bp	-kc2 -B8G -H4 -j48
	NA12878 2x151bp<br>NA24631 2x151bp<br>NA24143 2x250bp<br>NA24385 2x250bp	-kc2 -B50G -H4 -j48
MEGAHIT v1.2.9	NA12878 2x151bp<br>NA24631 2x151bp	–k-min 69 –k-max 141 –k-step 6 -t 48
	NA24143 2x250bp<br>NA24385 2x250bp	–k-min 99 –k-max 201 –k-step 6 -t 48
DISCOVAR de novo 52488	NA12878 2x151bp<br>NA24631 2x151bp<br>NA24143 2x250bp<br>NA24385 2x250bp	NUM_THREADS=48
SPAdes v3.15.3	*E. coli* 2x100bp<br>*E. coli* 2x150bp<br>*E. coli* 2x151bp	--isolate -t48
Unicycler v0.4.8	*E. coli* 2x100bp<br>*E. coli* 2x150bp<br>*E. coli* 2x151bp	-t48
QUAST v5.0.2	*E. coli* 2x100bp<br>*E. coli* 2x150bp<br>*E. coli* 2x151bp<br>*C. elegans* 2x110bp<br>*A. thaliana* 2x151bp	-t48
	NA12878 2x151bp<br>NA24631 2x151bp<br>NA24143 2x250bp<br>NA24385 2x250bp	--large -t48
BUSCO v5.2.2	*E. coli* 2x100bp<br>*E. coli* 2x150bp<br>*E. coli* 2x151bp	-m genome -l enterobacterales_odb10
	C. elegans 2x110bp	-m genome -l nematoda_odb10
	A. thaliana 2x151bp	-m genome -l brassicales_odb10
	NA12878 2x151bp<br>NA24631 2x151bp<br>NA24143 2x250bp<br>NA24385 2x250bp	-m genome -l primates_odb10
